# Photophysical study and *in vitro* approach against *Leishmania panamensis* of dicloro-5,10,15,20-tetrakis(4-bromophenyl)porphyrinato Sn(IV)

**DOI:** 10.12688/f1000research.52433.2

**Published:** 2021-07-01

**Authors:** Fabián Espitia-Almeida, Carlos Diaz-Uribe, William Vallejo, Doris Gómez-Camargo, Arnold R. Romero Bohórquez, Cristian Linares-Flores

**Affiliations:** 1 Grupo de Fotoquímica y Fotobiología, Universidad del Atlántico, Barranquilla, Colombia; 2 Grupo de Investigación UNIMOL, Universidad de Cartagena, Cartagena, Colombia; 3 Facultad de Ciencias Básicas y Biomédicas, Universidad Simón Bolívar, Barranquilla, Colombia; 4 Grupo de Investigación en Compuestos Orgánicos de Interés Medicinal (CODEIM), Parque Tecnológico Guatiguará, Universidad Industrial de Santander, Bucaramanga, Colombia; 5 Facultad de Ingeniería, Centro de Química Orgánica y Productos Naturales, Instituto de Ciencias Químicas Aplicadas, Universidad Autónoma de Chile, Santiago de Chile, Chile

**Keywords:** Photodynamic therapy, porphyrin, Leishmania panamensis, Photophysical study, in vitro, porphyrinato

## Abstract

**Background: **Photodynamic therapy activity against different biological systems has been reported for porphyrins. Porphyrin modifications through peripheral groups and/or by metal insertion inside the ring are main alternatives for the improvement of its photo-physical properties. In this study, we synthesized and characterized 5,10,15,20-tetrakis(4-bromophenyl)porphyrin and the dicloro-5,10,15,20-tetrakis(4-bromophenyl)porphyrinato Sn(IV).

**Methods:** Metal-free porphyrin was synthesized using the Alder method, while the Sn(IV)-porphyrin complex was prepared by combining metal-free porphyrin with stannous chloride in DMF; the reaction yields were 47% and 64% respectively. Metal-free porphyrin was characterized by UV-Vis, FT-IR, ESI-mass spectrometry and ^13^C-NMR. Additionally, the Sn(IV) -porphyrin complex was characterized using UV-Vis and FT-IR. Cyclic voltammetry tests in four different solvents. The fluorescence quantum yield (Φ _f_) was measured using fluorescein as a standard, the singlet oxygen quantum yield (Φ _D_) was estimated using the standard 5,10,15,20-(tetraphenyl)porphyrin (H2TPP) and the quencher of singlet oxygen 1,3-diphenylisobenzofuran (DPBF).

**Results:** UV-Vis assay showed typical Q and Soret bands for porphyrin and its metallo-porphyrin complex. Compounds showed photoluminescence at the visible range of electromagnetic spectrum. The inclusion of the metal in the porphyrin core changed the Φ _f_ from 0.15 to 0.05 and the Φ _D_ increased from 0.55 to 0.59. Finally, the effect of the compounds on the viability of *L. panamensis* was evaluated by means of the MTT test. The results showed that both compounds decreased the viability of the parasite; this inhibitory activity was greater under light irradiation; the porphyrin compound had IC _50 _of 16.5 μM and the Sn(IV)-porphyrin complex had IC _50 _of 19.2 μM.

**Conclusion:** The compounds were synthesized efficiently, their characterization was carried out by different spectroscopy techniques and their own signals were evidenced for both structures, both compounds decreased the cell viability of *L. panamensis*.

## Introduction

Porphyrins and metalloporphyrins are versatile macrocyclic organic compounds, from the structural viewpoint; the porphyrin main skeleton consists of four pyrrole rings bound through their alpha carbons (α-C) with four aldehydes. ^1, 2^ These structural characteristics confer on the porphyrins a variety of properties such as high conjugation, symmetry, and planarity. Additionally, they acquire the ability to complex with a large number of metals in their interior through the coordination of the four pyrrolic nitrogen atoms submerged in the molecule. ^3, 4^ The conjugated and aromatic structure of porphyrins allows interactions between π electrons and different metals, facilitating the binding to their coordination centers. ^5^ Porphyrins have absorption and intense electronic emission at wavelengths greater than 400 nm, small energy for HOMO-LUMO transitions and the ability to adjust their optical redox properties. ^6- 10^ All these properties make porphyrins relevant macromolecules in several chemistry fields (e.g. material science, optics, catalysis, transformation and storage of energy, medicine, pharmacology). ^11- 17^ In recent years, porphyrins have emerged as important promising photosensitizers in photodynamic therapy (PDT); porphyrin and its derivatives demonstrate great efficacy as both antibacterial and antiviral agents against different species due to its exceptional photodynamic properties. ^18, 19^ Porphyrins have potential applications in biological sciences, therefore, it is pertinent to develop simple synthetic pathways that lead to compounds with unique physical and chemical characteristics.

Nowadays different strategies to improve porphyrin photodynamic properties have been applied: (a) structural modification of the base ring with the addition of a variety of substituents at *messo* position, and (b) the inclusion of metals into the porphyrin core. ^20- 22^ The photophysical properties of porphyrins and their metallic-derivatives are affected for both the peripherally and/or axial substituents and the central metal into porphyrin core. ^23^ The porphyrins and their metallic-derivatives act efficiently as sensitizing agents and they have presented phototoxic activity against different pathogens, such as bacteria, fungi, viruses and parasites. ^24- 27^ Different porphyrinic photosensitizers have reported anti-leishmanicidal activity against *Leishmania tarentolae* in promastigote stage (ethyl and diethyl carbaporphyrin), against amastigotes of *L. panamensis*, and promastigotes of *L. major* and *L. braziliensis* (β-substituted porphyrinic systems). ^28- 32^ Improved pharmacological responses have been found when incorporating metals in the macrocycle, as reported by Gomes *et al.* when evaluating the activity against *L. amazonensis* against derivatives metalated with Bi (III) and Sb (IV) (IC50 of 93.8 μM and 52.4 μM respective). ^33^ Another Zn (II) metalated porphyrin derivative evaluated against promastigotes of *L. braziliensis* reduced parasite viability with greater efficiency than the metal-free derivative. ^34^ This type of modification alters the steric and electronic nature of porphyrins giving rise to new molecules that have specific and unique properties, and it also presents a promising alternative for modifying photophysical properties of the compounds: (a) quantum singlet oxygen performance, (b) the range of the therapeutic window, (c) photostability and (d) lipophilicity could be improved. ^35^

Although, *5,10,15,20-tetrakis(4-bromophenyl)-porphyrin* ( **compound 1**) and dicloro-5,10,15,20-tetrakis(4-bromophenyl) porphyrinato Sn (IV) ( **compound 2**) are commercially available, the reports about of its application as sensitizers in PDT are few. Therefore, in this study, we analyzed the photophysical behavior of ( **1**) and ( **2**) as regards their potential use in PDT against *Leishmania panamensis.*

## Methods

### Preparation and identification of compounds

**Figure 1.**
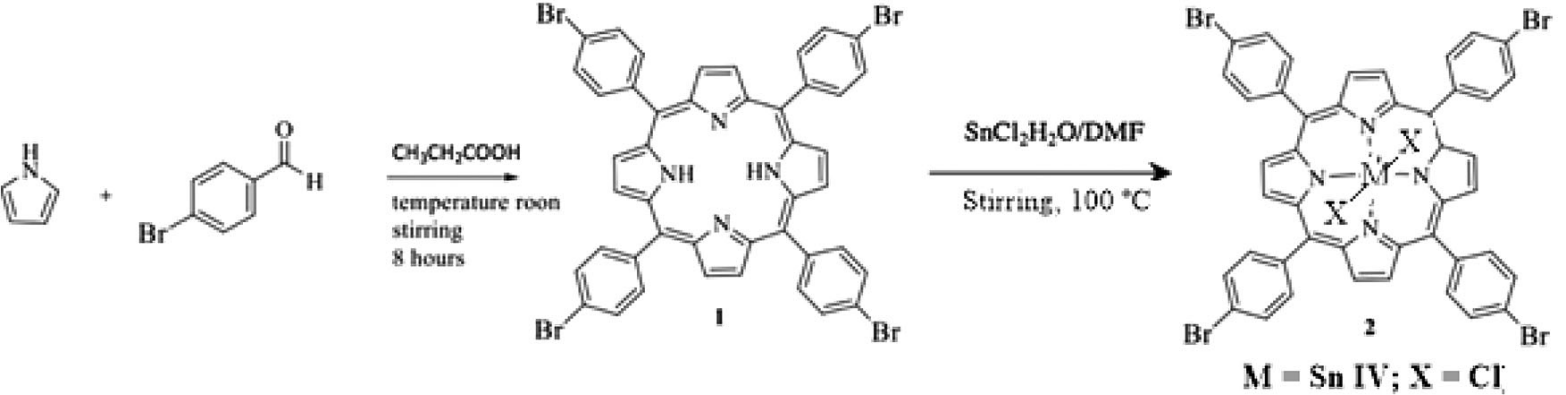
Synthesis route of compounds ( **1**) and ( **2**).

All reagents and solvents were purchased from Sigma Aldrich. We prepare the porphyrin 5,10,15,20-tetrakis(4-bromophenyl) porphyrin ( **1**) based on Adler’s method, ^36^ introducing a small modification that consisted of leaving the reaction for 8 hours at room temperature and stirring in an open container, using the oxidative power of oxygen to convert more of the chlorin by-product into porphyrin. In summary, equimolar amounts of pyrrole and 4-bromobenzaldehyde were mixed in propionic acid for 8 hours at ambient temperature under an atmosphere of air. Dicloro-5,10,15,20-tetrakis(4-bromophenyl) porphyrinato Sn (IV) ( **2**) was synthesized by porphyrin precipitation in metal chloride solicitation ( Figure 1). The formation of the final products was followed by thin layer chromatography on aluminum foil UV254 TLC, the mobile phase was petroleum ether-ethyl acetate (2:1). The compounds were characterized using ^13^C NMR spectra (Bruker AC-400 spectrometer); the ^13^C NMR chemical shifts are reported as ppm (δ), relative to CDCl _3_ (signal located at 7.29 ppm). Infrared spectrum was measured on the equipment ECO-ART alpha Bruker FTIR spectrometer. To perform UV-Vis spectrum (using a UV-2401PC UV-Vis spectrophotometer), we dissolved 2.0×10 ^−5^ g of each compound in ethyl acetate, and finally, we obtained the mass spectrum by dissolving the compound in methanol (using ESI-LC-MS/MS ion Trap amaZon, Bruker spectrometer). Furthermore, we measured fluorescence quantum yield (using a PTI um 40 fluorimeter) and singlet oxygen quantum yield, ^37^ and electrochemical characterization was performed in four different dissolvents (Dimethylformamide-DMF, dichloromethane -DCM, dimethylsulfoxide-DMSO, tetrahydrofuran-THF) containing 1.0×10 ^−1^ M tetrabutylammonium perchlorate ((C _4_H _9_) _4_N(ClO _4_), Aldrich 98% purity) as a supporting electrolyte for all electrochemical measurements.

Compound ( **1**) was prepared by mixing 10 mmol pyrrole and 10 mmol 4-bromobenzaldehyde in 80 ml propionic acid for 8 hours at ambient temperature in an open container. The product was extracted from the reaction medium by adding 60 mL cold methanol and filtering by gravity, the filtrate was dried at room temperature, obtaining 1.4136 g of a purple solid that was finally purified using column chromatography (mobile phase; ether-ethyl acetate 20:2). Yield: 47.28%; melting point > 300°C; UV-Vis (ethyl acetate) 414, 512, 546, 591, 645; FT-IR (cm ^−1^): N-H (3350), C=C (1470.28), C=N (1088.22), C-N (964.28); ^13^C NMR (CDCl _3_, 400 MHz): 118.86 (Ar), δ = 122.92 (Ar), δ = 130.22 (β-py), δ = 131.95 (Ar), δ = 135.95 (Ar *_ipso_*), δ = 140.91 (Ar-Br); (M+H) m/z = 930.9. These results concur with previous reports for this compound. ^37, 38^

Compound ( **2**) was prepared by mixing 0.5478 mmol of ( **1**) with 2.2164 mmol SnCl _2_.2H _2_O in DMF (80 mL) for 4 hours at ambient temperature, and by stirring. After that, we added cold water and TBrPP-Sn (IV) precipitated; this solid was washed and dried at ambient temperature. The compound was purified by column chromatography (mobile phase; petroleum ether-ethyl acetate 5:1). Yield: 64.4%; melting point > 300°C; UV-Vis: 426, 560, 600. These results concur with previous reports for this compound. ^37, 39^

### Photophysical properties

Fluorescence quantum yield (ϕ _f_) was determined by the comparative method in a PTI um 40 fluorimeter. Using as a standard fluorescein dissolved in water, porphyrin ( **1**) and metalloporphyrin ( **2**) was dissolved in ethyl acetate. All fluorescences were determined taking as excitation wavelength the maximum of the Soret band, using a 2 nm slit and a 420-750 nm scan. The fluorescence quantum yield was calculated with the following *eq. 1.* ^37, 40, 41^

(Eq. 1) $$\phi_{x}=\phi f_{est}*\frac{F_{x}*A_{est}*\eta_{est}^{2}}{F_{est}*A_{x}*\eta_{x}^{2}}$$

F _x_ and F _est_ are the areas under the fluorescence emission curve of compounds ( **1**), ( **2**) and the standard. A x and A are sample absorbances and standard at the excitation wavelength. ɳ x and ɳ are the respective refractive indices of the solvents (ethyl acetate; ɳ = 1.3724, water; ɳ = 1.33336).

Singlet oxygen quantum yield (Φ _∆_) of ( **1**) and ( **2**) was performed by the graphical method, using 1,3-diphenylisobenzofuran (DPDF) as singlet oxygen scavenger, and singlet oxygen generator standard 5,1,15,20-(tetraphenyl) porphyrin (H2TPP). The tests were carried out by preparing a 1×10-9 M solution of each compound in DMF in triplicate and calculated with *eq. 2.* ^37, 40, 41^

(Eq. 2) $$\phi \Delta =\phi \Delta_{\mathrm{standar}}*\frac{W}{W^{\mathrm{standar}}}$$

Where: Φ∆standard is the singlet oxygen quantum yield of the H2TPP standard in DMF (0.64). W and W _standard_ are the slopes of the degradation curves of the DPDF.

### Electrochemical characterization

Cyclic voltammetry (CV) data were recorded using a single-compartment electrochemical cell with a maximum electrolyte volume of 10 mL. A CH Instruments (Model 600E) Electrochemical Analyzer was used for the electrochemical measurements. The working electrode was a glassy carbon 3 mm in diameter on Teflon R (CH Instruments). The reference electrode used was an Ag ^+^/Ag electrode on Teflon R; we used a solution 1.0×10 ^−3^ M AgNO _3_ in electrolyte support; the auxiliary electrode was a platinum of 99.99% from CH Instruments. The electrochemical characterization was carried out using cyclic voltammetry and linear voltammetry. The peak current intensity $\;i_{p}\left[A\right]$ , in cyclic voltammetry, is given by the Randles-Sevcik *Eq.3*: ^42^

(Eq. 3) $$i_{p}=\left(2.69\times 10^{5}\right)n^{\frac{3}{2}}AD^{\frac{1}{2}}Cv^{\frac{1}{2}}$$

where n is the number of electrons in the redox reaction, $A\left[{\mathrm{cm}}^{2}\right]$ is the area of the working electrode, $\;D\left[{\mathrm{cm}}^{2}s^{-1}\right]$ is the diffusion coefficient for the electroactive species, $v\left[Vs^{-1}\right]$ is the scan rate, and $C\;\left[\mathrm{mol}\;{\mathrm{cm}}^{-3}\right]$ is the concentration of the electroactive species in the electrode. The anodic and cathodic peak currents are equal, and the ratio $i_{p,a}/i_{p,c}$ is 1.0. The half-wave potential, $E_{1/2}$ , is midway between the anodic and cathodic peak potentials, *Eq. 4.*

(Eq.4) $$E_{1/2}=\frac{E_{p,a}+E_{p,c}}{2}$$

### Biological assay

*Leishmania panamensis* (UA140) was used in *in vitro* tests for the evaluation of the leishmanicidal potential of the compounds ( **1**) and ( **2**). Leishmanicidal activity was determined as the ability of the compounds to decrease the viability of the parasite, for this the MTT method is widely used in the literature (MTT Assay Protocols, Thermo Fisher Scientific). Conditions were previously standardized by our working group. ^37, 43- 45^

### Parasite culture and viability using MTT assay

*Leishmania panamensis* (UA140) were cultured in RPMI-1640 supplemented with 10% fetal bovine serum, 1% glutamine and 1% antibiotics (200 U penicillin/200 μg Amikacin) under incubation conditions 5% CO _2_. ^37, 45^ The metacyclic promastigotes in the infectious stage were isolated from stationary cultures. The parasite viability was estimated by MTT assay. ^37, 43- 45^ Anti-leishmanicidal activity was evaluated at different concentrations (1, 10, 50, 100 and 200 μM) of compound and positive control (Glucantime), test compounds and positive control were dissolved in dimethyl sulfoxide (DMSO),working concentrations were obtained by adding 10 µL of compound in a final volume of 200 µL in each well of the microplate, under visible-light irradiation, at incubation times of 24, 48 and 72 hours. ^37^ We used 6 Omnilux LED bulbs located at a close chamber, and an incident photon flow per unit volume I _o_ was 5.7 × 10 ^−7^ Einstein*L ^−1^s ^−1^. Each trial was performed in triplicate. Plates were analyzed using SkanIt software. We applied an ANOVA test to determine the differences or similarities between treatments and positive control. In addition, a post hoc analysis was performed using Tukey statistics. Finally, differences were considered to be significant when *p < 0.05.*

## Results and discussion

### UV-Vis assay

**Figure 2.**
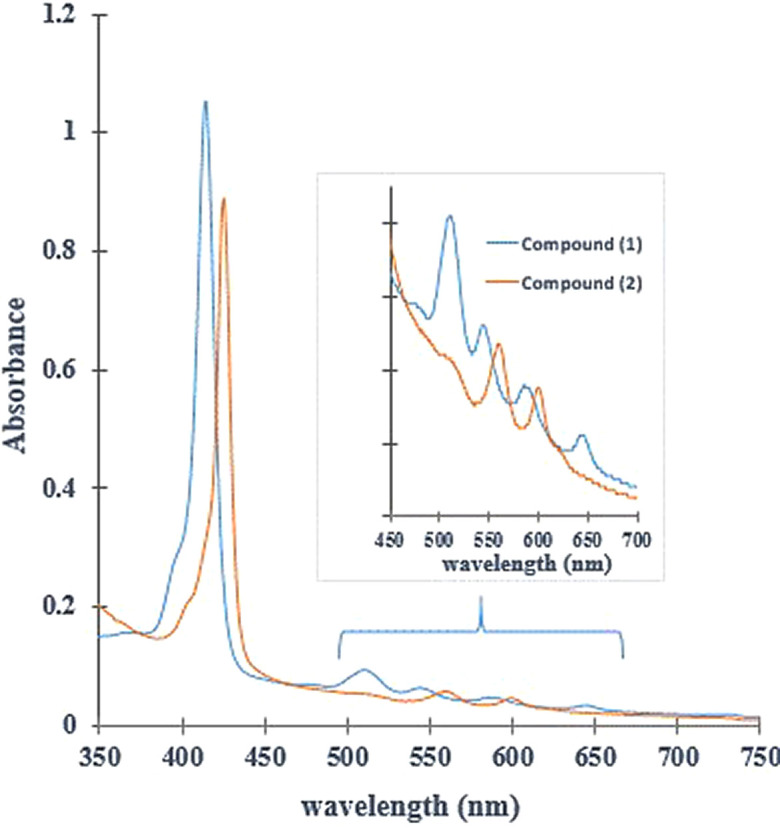
UV-Vis spectrum ( **1**) and ( **2**). The bands α and β represent the Q band in the porphyrin Sn (IV) complex ( **2**).

The UV-Vis spectrum of ( **1**) ( Figure 2), shows a band of maximum absorption located at 414 nm (Soret band), generated by a _1u_(π)-e _g_^*^(π) transitions and four lower absorption Q band located at 515nm, 547nm, 588nm and 645 nm, which corresponds to a _2u_(π)-e _g_^*^(π) transitions. ^46, 47^ The UV-Vis spectrum of compound ( **2**) shows one Soret band and only two Q bands. When the Sn (IV) ion coordinates nitrogen atoms inside the porphyrin ring, the porphyrin symmetry increases. Furthermore, the reduction in the number of Q bands indicates that the metal effectively entered the macrocycle. ^48^ The intensity of the Q bands is correlated with the relative stability of the metalloporphyrin: when the signals are of low intensity, the metallocomposites are highly stable and their atoms are located in the square plane. ^49, 50^ Ohsaki *et al.* reported a similar change in the UV-Vis spectrum after tin (IV)-insertion into the porphyrin core synthesized in water at ambient temperature. ^51^ Moreover, the Soret band for ( **2**) had red shift from 414 nm to 425nm (near to 11 nm). The direct coordination between Sn (IV) ion and porphyrin core could extend conjugation from porphyrin to metal ion; in this case, the electronic excitation will require lower energy absorption due to increasing conjugation–this process requires longer wavelength than pure porphyrin. ^10, 52^ Figure 2 shows that ( **1**) and ( **2**) have photo-activity inside window 400 to 7000 nm. Although the compounds do not have a considered absorption in the red rank of the visible light spectrum from 600 nm to 800 nm (this radiation can reach a penetration depth of 8.0 mm inside tissue), they absorb radiation inside range 500-600 nm. This radiation penetrates approximately 4.0 mm, and such penetration capacity is suitable for potential application in cutaneous treatments. ^53^

### Photophysical properties

*Deamination of Փ _f_*

**Figure 3.**
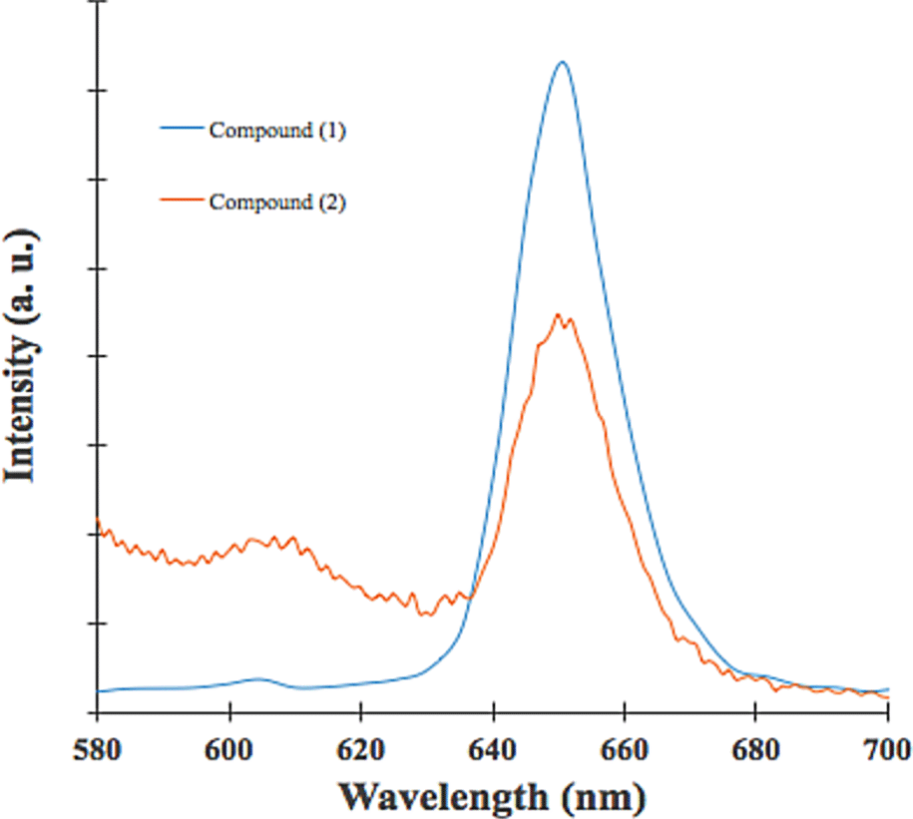
Emission spectra of (1) and (2).

Information related to the efficiency of fluorescence emission is important to explain the inactivation pathway related to PDT. ^54^ Figure 3 shows fluorescence emission for ( **1**) and ( **2**); both show photoluminescence at a visible range in the electromagnetic spectrum. As shown in Figure 3, fluorescence emission wavelength was located at 651 nm for both compounds, the energy transition did not change after the Sn (IV) ion insertion; however, fluorescence emission intensity was more high compound ( **1**). This effect is due to the Sn (IV) ions insertion inside the porphyrin core decreases significantly fluorescence effect. As shown in Table 1, the Φf of ( **1**) was three times greater than ( **2**); the Sn (IV) ions inside the porphyrin core could increase the non-radiative decay of the excited singlet state of porphyrin, ^37^ Sn (IV) ions within the porphyrin nucleus could increase disintegration by inter-system crossover (ISC), and this pathway is governed by orbital spin coupling at the central atoms; in this case, the insertion of Sn (IV) reduces the fluorescence emission. ^9, 55, 56^ A similar effect was reported for cupper insertion inside *meso*-porphyrinic complexes. ^56, 57^

**Table 1.** Photophysical properties ( **Փ**_**f**_ y **Փ**_**Δ**_) of ( **1**) and ( **2**).

Compound	λ _abs_					λ _em_	Φ _f_	Φ _Δ_
	Soret Band	Q Bands						
**(1)**	414	512	546	591	645	651	0.15 ± 0.01	0.55 ± 0.03
**(2)**	426	560	594	---	---	651	0.05 ± 0.01	0.59 ± 0.04

*Deamination of Փ _Δ_*

**Figure 4.**
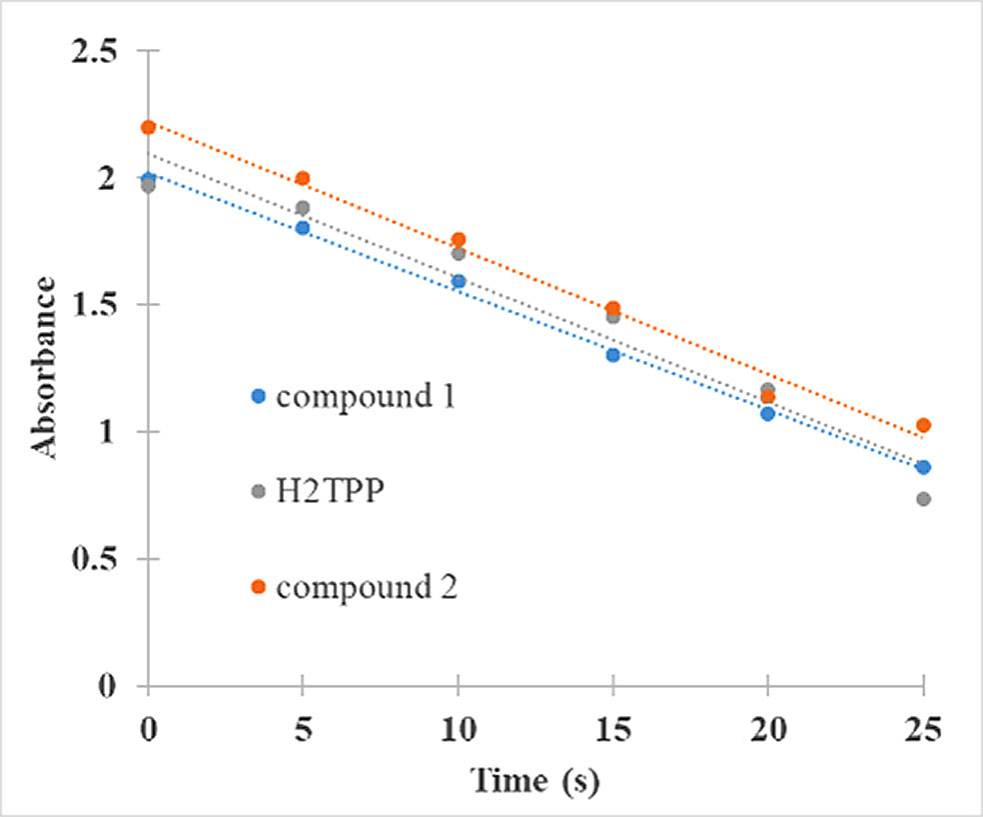
Absorbance and degradation of DPBF at 415 nm as a function of reaction time with ( **1**), ( **2**) and standard. Linear fit is also shown.

The generation of singlet oxygen produced by ( **1**) and ( **2**) was quantified by chemical entrapment using DPBF. To estimate the Φ _Δ_, the degradation of DPBF was measured at 415 nm over time ( Figure 4), the lower the absorbance, the greater the degradation of DPBF mediated by singlet oxygen. The complex Sn (IV)-porphyrin ( **2**) presented higher Φ _Δ_ compared to ( **1**) ( Table 1), the difference in Φ _Δ_ between the compounds is 7%, and this difference is directly attributed to the insertion of the metal ion Sn (IV) inside the macrocycle. Sn (IV) would be generating greater stability of the triplet state of the molecule and improving the interaction with molecular oxygen, which is reflected as a greater translocation of the molecular oxygen spin and its subsequent conversion into singlet oxygen. ^4, 59- 62^ These sensitizers show promise in PDT for its Φ _Δ_ values, and could in the future be candidates in clinical trials like its counterpart Lutetium Texaphyrin, which has a Φ _Δ_ as low as 0.11. ^63^

### Electrochemical characterization

**Figure 5.**
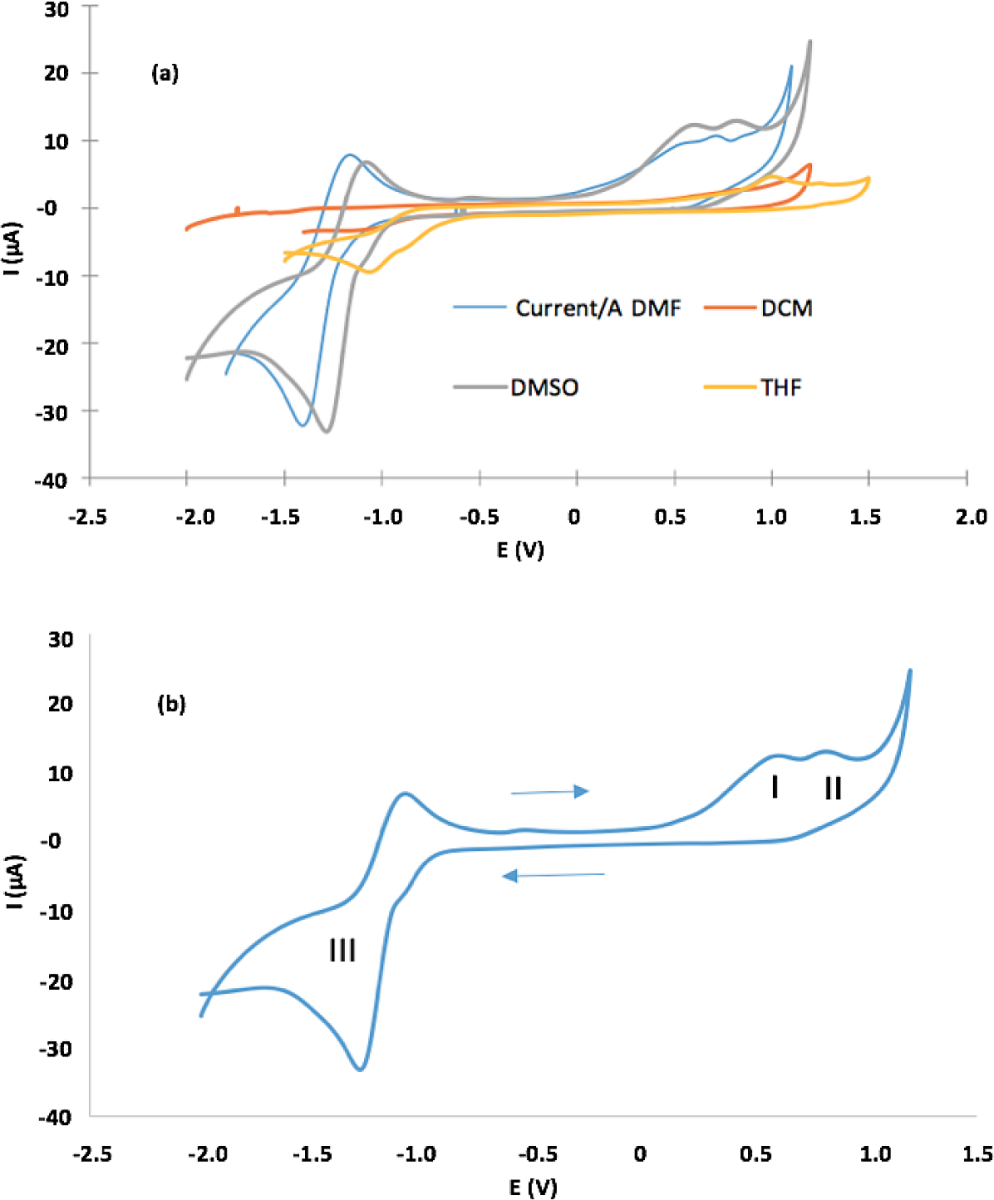
**a**) CVs of the Sn (IV)-porphyrin complex in DMF, DCM, DMSO and THF (0.1 M of TBAClO _4_. Scan rate = 0.1 V/s). **b**) CVs (in detail) for oxidation and reduction of the Sn (IV)-porphyrin complex in DMSO.

The biological systems present microheterogeneity, caused by the coexistence of microphases such as aqueous polar and highly hydrophobic lipid. ^64, 65^ Therefore it is relevant to study the physicochemical properties of sensitizers at different media. We have studied the electrochemical behavior of ( **2**) using cyclic voltammograms (CV) in four different solvents. Figure 5 shows CVs and Table 2 lists electrochemical parameters. Porphyrin had irreversible one-electron oxidation at E _pa_, which varies between 0.55 V for DMF and 1.0 V for THF, in addition to one quasi-reversible reduction peak between −1.01 V for THF and −1.41 for DMF ( Figure 5a). The latter is clearer when DMF and DMSO are solvents. CVs (in detail) in DMSO, presenting three redox processes: ( **a**) oxidation processes (I and II) related to the formation of monocationic and dicationic porphyrin spaces, ( **b**) (a) oxidation processes (I and II) related to the formation of monocationic and dicationic porphyrin spaces, (b) reduction process (III) that results in the formation of the anionic porphyrin species ^66^ ( Figure 5b).

**Table 2.** Determination of half-wave potentials for four different solvents.

Solvent	Oxidation		Reduction First	ΔE _1/2_
	Second	First		
DMF	0.71	0.54	−1.41	0.98
DCM	–	0.83	−1.11	0.97
DMSO	0.81	0.59	−1.27	0.93
THF	1.25	1.00	−1.01	1.01

The conformational and structural changes observed in reversible electron transfer reactions can be examined through the potential differences between the first and second oxidation states. ^46, 67^ Table 2 shows the oxidation and reduction potentials. The couple III presented an almost reversible behavior accompanied by a separation of anodic-cathodic peaks ΔEp> 60 mV, in addition, it presented an anodic-cathodic peak potential ratio of approximately unity (1.0). This result is characteristic of a nearly reversible monoelectronic process. The electron-attractant character of the bromine substituents could be significantly influencing the electrochemical properties of the derivatives ( **1**) and ( **2**). ^68, 69^ Likewise, Table 2, shows a slight effect on ΔE _1/2_ by change of dissolution solvent. THF had the highest ΔE _1/2_ due to lower dielectric constant value (ε), and this solvent had smaller dielectric constant value (ε _DMSO_ = 46.70, ε _DMF_ = 36.70, ε _DCM_ = 8,93 and ε _THF_ = 7.58) between all aprotic dipolar solvents under study herein. Our data on the potentials for oxidation and reduction of TBrPP-Sn (IV) ( Table 2) are consistent with previous reports published in the literature on metalloporphyrin-like compounds. ^70, 71^

**Figure 6.**
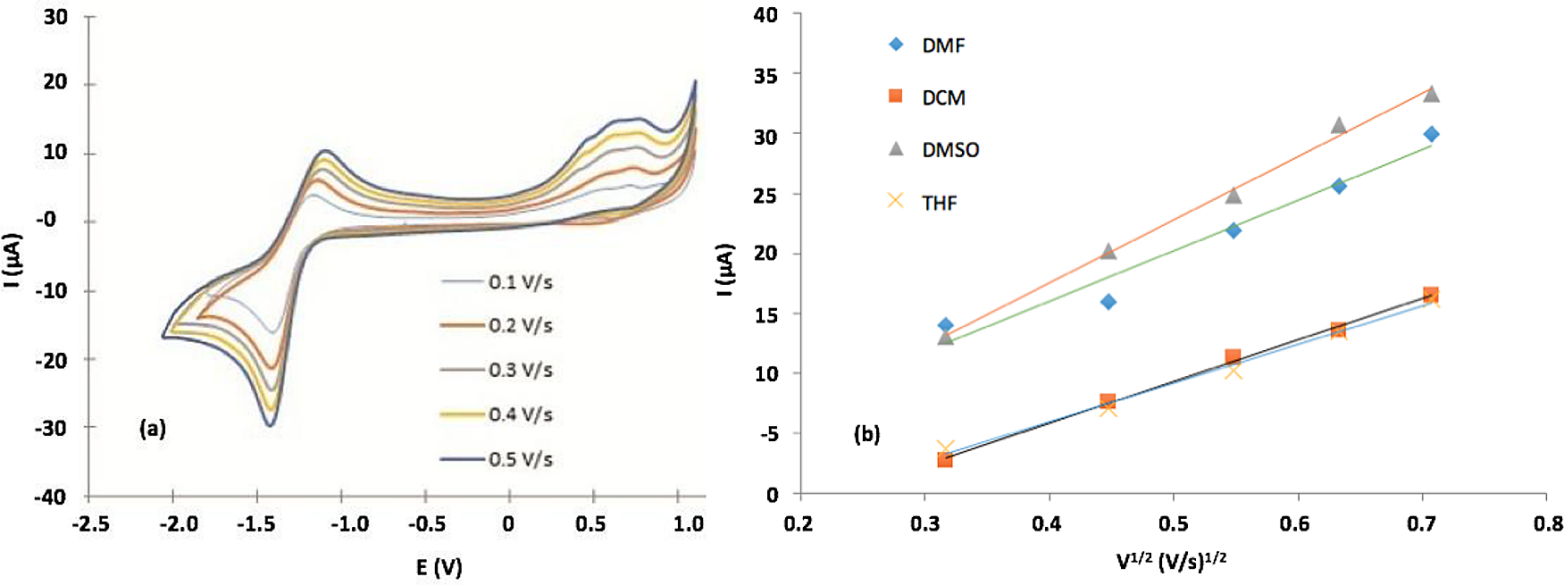
**a**) CV measured at scanning speeds of 0.1-0.5 Vs ^−1^ for a 1×10 ^−3^ M solution of the Sn (IV) -porphyrin complex in DMF solvent (0.1 M TBAC104). **b**) Estimation of the relationship between the maximum current and the square root of the scanning speed.

We studied the scanning speed effect on the current response of CVs for each solvent to determine if the redox process was controlled by diffusion or by adsorption. Figure 6a shows Cvs for ( **2**) at different scanning speeds in DMF. Figure 6a shows an anode peak for all scan rates in the range 0.8-1.3 V for all solvents used. The relationship found between the scanning speed and the peak current was directly proportional with linear increase, and the peak potential anodically shifted; additionally, when the scanning speed was increased, the peak became broader. Figure 6b shows that the peak current correlated with the square root of the scan rate for each solvent studied. Table 3 shows R ^2^ and linear equation fitting for each test. The fitting results indicated that the process is controlled by diffusion, then hydrodynamics of media (e.g. polarity, density, viscosity) determines the redox process rate. ^66- 68^ Furthermore, the linear fit of the line plot of Ip versus *v* ^*1*/2^ indirectly indicates a relationship between the diffusion coefficient and DMSO; and DMF had the highest slope value, suggesting that the diffusion coefficient for these solvents was greater than the diffusion coefficient for THF and DCM. This result is associated with the value of the dielectric constant, as discussed. ^67, 68^

**Table 3.** Fitting parameters for I _p_ versus v ^1/2^ plot to each solvent.

Solvent	Linear equation	R ^**2**^	slope _**)**_ (μAV ^**-1/2**^s ^**1/2**^)
**DMF**	y=4.22×10 ^−5^x *−*8.1×10 ^−7^	0.956	4.22
**DCM**	y=3.47×10 ^−5^x *−*8.1×10 ^−6^	0.997	34.7
**DMSO**	y=5.29×10 ^−5^x *−*3.6×10 ^−6^	0.995	52.9
**THF**	y=3.21×10 ^−5^x *−*6.9×10 ^−6^	0.992	32.1

**Figure 7.**
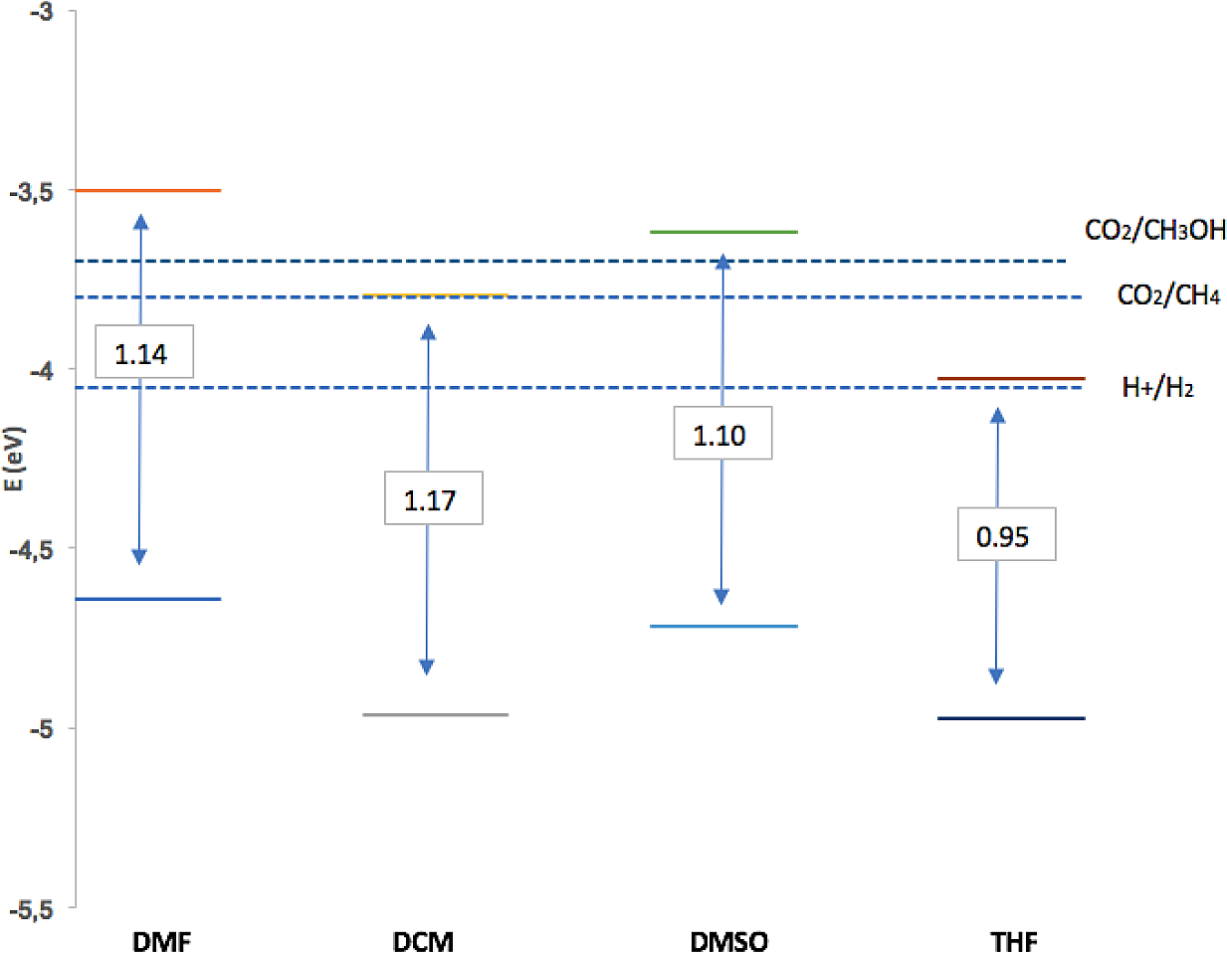
Band gap and band edge positions to Sn-Porphyrin derivative for dissolvent used in this work. Y-axis energy levels for the reduction and oxidation potentials of the dissociation reactions. pH = 7 y room temperature.

Figure 7 shows dissolvent effect on electrochemical band gap value ( **2**) for solvents studied in this work. The data obtained for the redox potentials (pH = 7.0 and room temperature) of the water separation reaction and the carbon dioxide reduction reactions to produce methane and methanol: Potential per redox couple; E (H2O/O2) = −5.26 eV; E (H +/H2) = −4.03 eV; E (CO2 / CH4) = −3.79 eV; E (CO2/CH3OH) = −3.65 eV. ^72^ It is evident that the value of the electrochemical band gap depends on the polarity of the solvent, ( **2**) had the smallest defective gap in THF. A requirement for the photosensitizer in PDT is the band gap; electrochemical characterization indicates that it is appropriate and suitable. Finally, the potentials described in Table 2 corresponding to the redox process of ( **2**) are in agreement with other reports for processes based on rings in porphyrin complexes. ^72, 73^

### Biological assay

A wide variety of molecules have been evaluated as possible therapy against *Leishmania* spp in recent years: (i) aluminum and zinc phthalocyanines (ii) methylene blue, 5-aminolevulinic acid and porphyrin. The field of research on new substances with challenging properties in medicine and pharmacology is an important topic, which has become more relevant due to the appearance of resistant and emerging microorganisms. ^74- 76^ The compounds ( **1**) and ( **2**) have been used to evaluate their effects on the viability of *L. panamensis* using the MTT method. The results are presented as cell viability of *L. panamensis* after exposure to different concentrations of ( **1**) and ( **2**) during incubation periods of 24, 48 and 72 hours, in darkness and under irradiation. The same procedure was done for the positive control (Glucantime).

**Figure 8.**
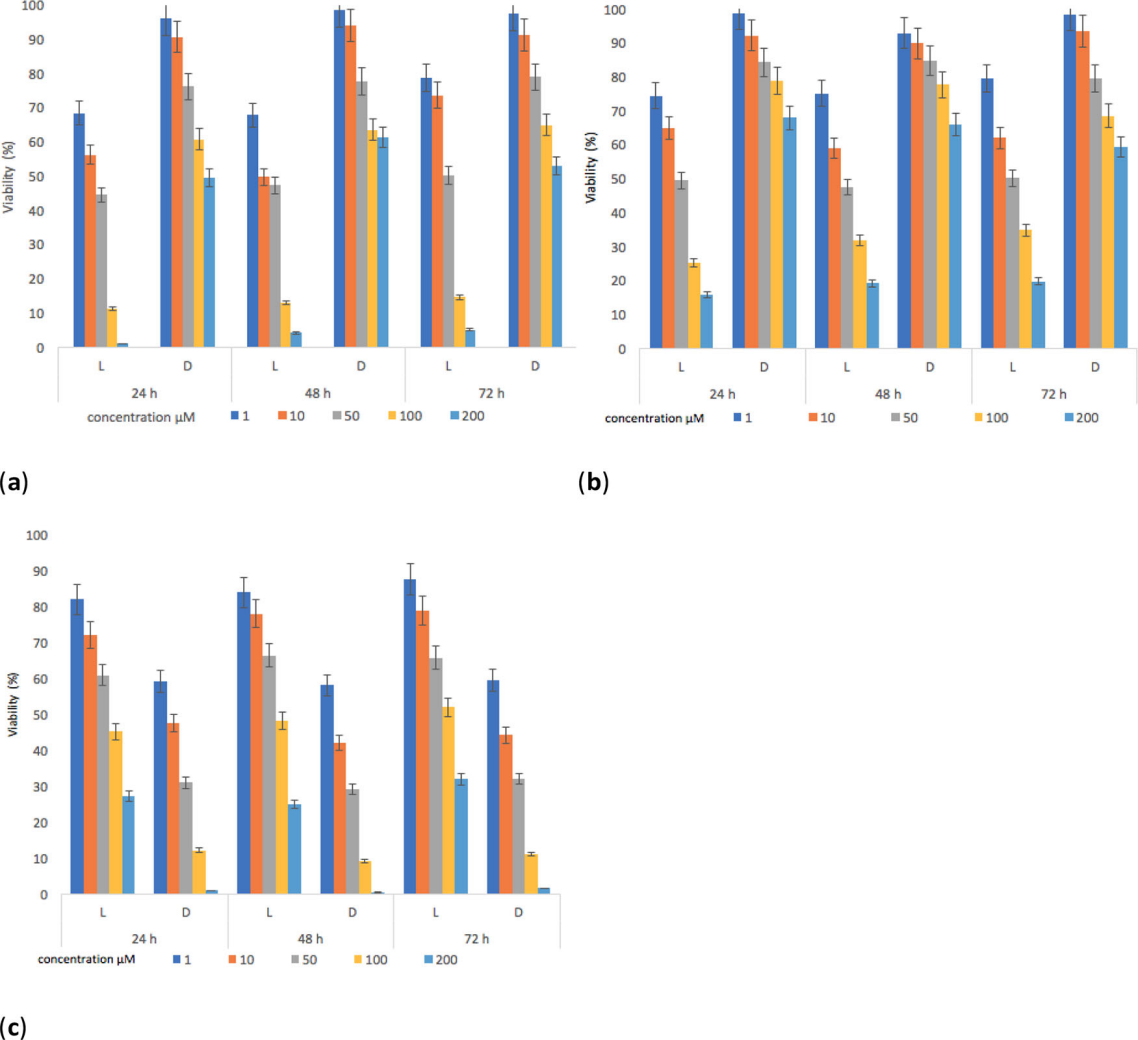
Parasite viability percentage results for *L. panamensis* against: ( **a**) compounds ( **1**), ( **b**) compounds ( **2**) and ( **c**) positive control drug with incubation periods of 24, 48 and 72 hours.

The results show that ( **1**) and ( **2**) presented inhibitory activities on parasite viability ( Figure 8). The decrease in the viability of *L. panamensis* was observed to a greater degree on the irradiated tests, this is due to the increased interaction capacity of the test compounds with oxygen, which induced the production of singlet oxygen. ^77, 78^

The contrary effect was evidenced for the reference standard, which reached better inhibitory activities on parasite viability in darkness. The highest effect leishmanicide was observed when the parasite was exposed to light for 24 hours and with concentrations of the compounds higher than 100 μM. Furthermore, the irradiation time ( 48– 72) had no significant effect on viability (%) results compared to samples irradiated for 24 hours. The IC _50_ ( Table 4) was determined using data found in the 24-hour test. Under irradiation, the IC _50_ of both compounds was lower compared to the positive control (Glucantime), with values of 16.5 and 19.5 μM respectively. The activation of sensitizers by light action ensures lower IC _50_ values. Besides, in the absence of light, the response was lower, thus being in line with findings of other reports. ^78^ Our results also show that ( **1**) and ( **2**) cause damage to the parasite, decreasing its survival rate by 1.5 times compared to the standard, which could induce a reduction in the healing time of a lesion.

**Table 4.** IC _50_ values for compounds TBrPP, TBrPP-Sn (IV) and positive control against *L. panamensis,* 24-hour incubation treatments.

compound	CI _**50**_ (μM) ± SD	
	Light	Darkness
**(1)**	16.5±0.1	105.0±0.3
**(2)**	19.0±0.1	> 200
**Gluc**	56.1±0.1	8.8±0.1

## Conclusions

In the present study, we synthesized and characterized ( **1**) and ( **2**). Compound structures were confirmed by spectroscopic techniques (UV-Vis, FT-IR, ^13^C- NMR and ESI-mass). Sn (IV) ion insertion inside in the porphyrin core reduced significantly Փ _f_ 0.15 to 0.05. Furthermore, Φ _Δ_ increased from 0.55 to 0.59 after metal insertion inside the porphyrin core. Electrochemical results showed that electrochemical properties were affected by the solvent dielectric constant, where THF had the highest ΔE _1/2_ due to a lower dielectric constant value. Moreover, the electrochemical assay showed a quasi-reversible reduction peak between −1.01 V in THF and −1.41 in DMF. The inhibitory results shown that ( **1**) and ( **2**) presented inhibitory activities on parasite viability. The highest inhibitory activity on the parasite was observed when the treatments were irradiated for 24 hours and with concentrations of the compounds of 200 μM, strengthening the hypothesis that parasite mortality is mediated by reactive oxygen species (especially singlet oxygen). These compounds were synthesized with low-cost methods and with acceptable synthesis yield, a fundamental aspect in the search for sensitizer candidates. The results of biological activity suggest these compounds could be applied in future applications of *in vivo* models as potential sensitizers of photodynamic therapy.

## Data availability

### Underlying data

Mendeley Data: Complementary material, http://dx.doi.org/10.17632/h2vmrdz4sg.1. ^79^

This project contains the following underlying data:

- 1. UV-Vis compound ( **1**) and ( **2**).xlsx [UV-Vis spectrum of compound 1 and 2]
- 2. Gráficos, RENDIMIENTO CUÁNTICO DE FLUORESCENCIA.xlsx [Graphics, Quantum fluorescence yield of compound 1 and 2]
- 2.1 Calculo de Qf.xlsx [Calculation of Quantum fluorescence yield (Qf) of compound 1 and 2]
- 3. 1. Compound 1 (DMSO). Rendimiento Cuántico Oxígeno Singulete – copia.xlsx [Calculation of Quantum Yield Oxygen Single of Compound 1 in DMSO]
- 3.2. Compound 2 (DMSO). Rendimiento Cuántico Oxígeno Singulete.xlsx [Calculation of Quantum Yield Oxygen Single of Compound 2 in DMSO]
- 4. ESI-MS, Compound 1.jpg [Mass spectrum of Compound 1 in methanol]
- 5. FT-IR, Compound 1.pdf [FT-IR spectrum of Compound 1]
- 6. RMN 13C, Compound 1.mnova [RMN 13C spectrum of Compound 1 in CDCl3]
- 7. Assay Biologoly_Compound 1 and 2_Luz vs L. Panamensis_Promastigote.xlsx [Assay Biological of Compound 1 and 2 in the presence of light against L. Panamensis promastigote]

Data are available under the terms of the Creative Commons Attribution 4.0 International license (CC-BY 4.0).
