# Supplementary material for: Photophysical study and in vitro approach against Leishmania panamensis of dicloro-5,10,15,20-tetrakis(4-bromophenyl)porphyrinato Sn(IV)
Source: F1000Res. 2021 Nov 8;10:379. Originally published 2021 May 12. [Version 3] doi: 10.12688/f1000research.52433.3 (PMC8581593; doi:10.12688/f1000research.52433.3)
Supplement: Peer review history

# Peer review: Reviewer response for version 3

**DOI:** 10.5256/f1000research.78968.r99462

**Published:** 2021-11-10

**Authors:** Martha Simões Ribeiro

**Affiliations:** 1 Center for Lasers and Applications, Nuclear and Energy Research Institute (IPEN-CNEN), São Paulo, Brazil

No further comments. The authors revised the manuscript properly.

Is the work clearly and accurately presented and does it cite the current literature?

Partly

If applicable, is the statistical analysis and its interpretation appropriate?

No

Are all the source data underlying the results available to ensure full reproducibility?

Partly

Is the study design appropriate and is the work technically sound?

Yes

Are the conclusions drawn adequately supported by the results?

Partly

Are sufficient details of methods and analysis provided to allow replication by others?

Partly

Reviewer Expertise:

Optical therapy

I confirm that I have read this submission and believe that I have an appropriate level of expertise to confirm that it is of an acceptable scientific standard.

# Peer review: Reviewer response for version 2

**DOI:** 10.5256/f1000research.57235.r93076

**Published:** 2021-09-22

**Authors:** Martha Simões Ribeiro

**Affiliations:** 1 Center for Lasers and Applications, Nuclear and Energy Research Institute (IPEN-CNEN), São Paulo, Brazil

This work aimed to verify the photodynamic activity of 5,10,15,20-tetrakis(4-bromophenyl)-porphyrin and dicloro-5,10,15,20-tetrakis(4-bromophenyl) porphyrinato Sn (IV) on promastigotes of *Leishmania panamensis* after porphyrin synthesis and characterization.

 Some issues should be addressed by the authors:

- Please, report the initial inoculum of parasites and the type of microwell plate.
- Please, report the emission of the light source. I am not sure what it means by visible light.
- Please, provide the light dose. Literature commonly uses J/cm^2.
- I am not sure if parasites were irradiated by 24, 48 and 72 h or if they were incubated by those times. Please, clarify.
- Authors report that figure 2 shows the photoactivity of the compounds between 400 and 7000 nm. Please, change 7000 for 700 nm.
- Please add another reference for information regarding light penetration in the skin. Ref 53 is not appropriated. My suggestion is to look for Steven Jacques or Valery Tuchin's papers.
- Please, add information regarding I, II, and III in the legend of figure 5 b.
- The irradiation impaired the glucantime antileishmanial effect. Do authors have a hypothesis for this finding?
- Authors should compare parasite metabolic activity following compound 1 and compound 2-mediated photodynamic treatment. Statistical analysis is necessary. It seems to me that compound 1 is significantly better than compound 2. However, compound 2 presented a higher singlet oxygen quantum yield. The authors should discuss this issue.
- I think the last sentence of the manuscript before conclusions is not supported by the authors' findings. Indeed, the authors assumed parasite damage since they do not show any image. However, MTT measures metabolic activity and its sensitivity is not high. Besides, glucantime in the dark promoted similar inactivation. Please, rephrase.

Is the work clearly and accurately presented and does it cite the current literature?

Partly

If applicable, is the statistical analysis and its interpretation appropriate?

No

Are all the source data underlying the results available to ensure full reproducibility?

Partly

Is the study design appropriate and is the work technically sound?

Yes

Are the conclusions drawn adequately supported by the results?

Partly

Are sufficient details of methods and analysis provided to allow replication by others?

Partly

Reviewer Expertise:

Optical therapy

I confirm that I have read this submission and believe that I have an appropriate level of expertise to confirm that it is of an acceptable scientific standard, however I have significant reservations, as outlined above.

# Peer review: Reviewer response for version 2

**DOI:** 10.5256/f1000research.57235.r88766

**Published:** 2021-07-15

**Authors:** John Mack

**Affiliations:** 1 Institute for Nanotechnology Innovation, Department of Chemistry, Rhodes University, Makhanda, South Africa

The revisions appear to be acceptable.

Is the work clearly and accurately presented and does it cite the current literature?

Partly

If applicable, is the statistical analysis and its interpretation appropriate?

Yes

Are all the source data underlying the results available to ensure full reproducibility?

Yes

Is the study design appropriate and is the work technically sound?

Yes

Are the conclusions drawn adequately supported by the results?

Yes

Are sufficient details of methods and analysis provided to allow replication by others?

Partly

Reviewer Expertise:

I carry out research that is relevant to all aspects of this study.

I confirm that I have read this submission and believe that I have an appropriate level of expertise to confirm that it is of an acceptable scientific standard.

# Peer review: Reviewer response for version 1

**DOI:** 10.5256/f1000research.55710.r85271

**Published:** 2021-05-24

**Authors:** John Mack

**Affiliations:** 1 Institute for Nanotechnology Innovation, Department of Chemistry, Rhodes University, Makhanda, South Africa

The manuscript describes the synthesis and further characterization of a Sn(IV) tetraarylporphyrin complex including its photosensitizer properties *and in vitro* studies of its activity against *L. panamensis.* The work has been carried out competently for the most part. The results are very much in line with expectations but are of sufficient interest in the context of the effect of the porphyrins on the viability of *L. panamensis* to merit publication on a platform like F1000. I recommend approval after minor revisions. I urge the authors to carefully consider the following points while preparing their revised manuscript:

 (a) The references should be brought up to date where appropriate. They appear to stop at around 2018 or so.

 (b) Were DMSO stock solutions used to solubilize the dyes for the *in vitro* studies? If so, the details must be provided.

 (c) Were filters used for the fluorescence measurements in Figure 3 and was a solvent baseline subtracted. The baseline should not rise like it does for compound 2. If not, the fluorescence quantum yields will have to be recalculated.

 (d) Can Figure 1 be redrafted in Chemdraw?

 (e) Decimal points should be used in the y-axis labels of Figure 2.

 (f) There is no need for "(u.a.)" in the y-axis title of Figure 4. Having absorbance values > 2 is problematic in this context in terms of the linear range of the spectrometer used, but if reasonable linearity was obtained there is probably no need to repeat the measurements.

 (g) The phrase "have photo-activity inside window 400 to 7000 nm" needs to be fixed.

 (h) The λ _abs_ values for the Q bands should also be provided in Table 1. λ _em _would normally be preferred rather than λ _emi_.

 (i) The authors should carefully re-evaluate whether their use of three significant figures in Table 2 is justified.

Is the work clearly and accurately presented and does it cite the current literature?

Partly

If applicable, is the statistical analysis and its interpretation appropriate?

Yes

Are all the source data underlying the results available to ensure full reproducibility?

Yes

Is the study design appropriate and is the work technically sound?

Yes

Are the conclusions drawn adequately supported by the results?

Yes

Are sufficient details of methods and analysis provided to allow replication by others?

Partly

Reviewer Expertise:

I carry out research that is relevant to all aspects of this study.

I confirm that I have read this submission and believe that I have an appropriate level of expertise to confirm that it is of an acceptable scientific standard, however I have significant reservations, as outlined above.
